# Perception of Master of Public Health Students regarding E-Learning in Covid-19 Era: A New Normal in Lower- and Middle-Income Countries

**DOI:** 10.12669/pjms.38.3.4626

**Published:** 2022

**Authors:** Saima Aleem, Naheed Mahsood, Zeeshan Kibria, Rubeena Gul

**Affiliations:** 1 Saima Aleem, BDS, MPH, FRSPH. Assistant Professor, Public Health, Sarhad Institute of Health Sciences, Peshawar, Pakistan; 2 Naheed Mahsood, MBBS, MPH, MHPE. Assistant Professor, Medical Education, Khyber Girls Medical College, Peshawar, Pakistan; 3 Zeeshan Kibria, MBBS, MPH, MHR. Deputy Director (ORIC) Khyber Medical University, Peshawar, Pakistan; 4 Rubeena Gul, MBBS, MCom Health. Associate Professor Community Medicine Khyber Medical College, Peshawar, Pakistan

**Keywords:** Covid-19, E-learning, Perception, Public health

## Abstract

**Objectives:**

To explore the perception of postgraduate public health students regarding e-learning in context to Covid-19 pandemic and its effect on their academic performances

**Methods:**

This cross-sectional study was conducted at Sarhad Institute of Health Science, SUIT Peshawar from 3^rd^ October 2020 to 4^th^ February 2021. The Census method was incorporated for sample selection. Participation in the study was subjected to consent by participants. A self-administered questionnaire was used for data collection. Data was analyzed using SPSS Version-26.

**Results:**

Out of 95 participants, 72 (75.8%) were males and 23 (24.2%) were females. The mean grade point average (GPA) of previous semester-1 and semester-2, when they were having a conventional education system on campus before the pandemic was 2.741±0.499 and 2.643±.498 respectively. The current mean GPA of semesters 1, 2, and 3 who had online classes during this pandemic was 2.41±0.66, 3.06±0.51, and 2.80±0.47 respectively. Fifty-one (53.7%) students preferred to use mobile for e-learning. Convenience to the use-learning management system (LMS) was 67.4% and 72.6% responded that their academic performance was positively affected by e-learning. Logistic regression revealed that source of learning (*p* 0.99), uninterrupted internet (*p* 0.87), convenience with LMS (*p* 0.17), stress (*p* 0.505), convenient communication with faculty (*p* 0.69), and compatibility with professional routine (*p* 0.21) were not significantly associated with good academic performance, however, students of semester 2 (*p* 0.001) and those using laptops (*p 0.02)* were more likely to get a GPA of 3.0 or above

**Conclusions:**

Students in this study had a positive perception regarding e-learning however, there is a definite need to amalgamate both online and on-campus learning modalities for post-graduate students especially during the uncertain situations.

## INTRODUCTION

In recent times, the coronavirus has emerged not only as a threat but a challenge to all the health systems and governments across the globe. It was more life-threatening to human life than any other lethal warfare. With the emergence of cases of covid-19 in China, the World Health Organization published a comprehensive document for countries, covering topics related to the management of an outbreak of a new disease on 12^th^ Jan 2020 but it was on 30^th^ Jan 2020 when WHO declared the novel coronavirus outbreak a public health emergency of international concern.^1^ Undoubtedly, Covid-19 emerged as the worst pandemic in human history not only in terms of mortality but by leaving the governments especially that of low- and middle-income countries (LMICs) helpless on all fronts. In response to Covid-19, the Pakistan government decided to halt all the daily life activities, and institutions were immediately closed. The education sector was no different and it was March 13, 2020, when all educational institutions were closed.^2^ Later, an online system was introduced as per directives of HEC to continue all educational activities.

**Fig.1:**
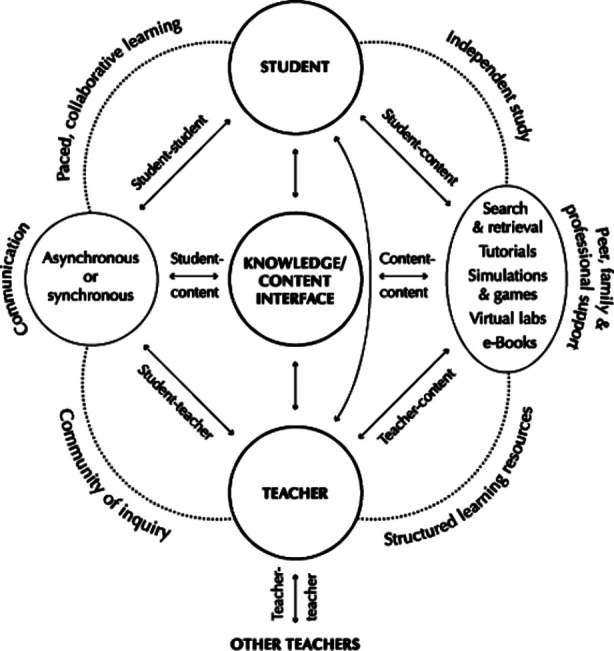
Anderson’s Model of E-Learning.^8^

Online education is an extension of distance learning incorporating both technical devices including computers, cell phones, laptops, and the internet^3^ but three terms distance learning, e-learning, and online learning are interconnected^4^. The relationship between online learning, e-learning, and distance learning can be established from Fig.1 for better understanding.

Globally, e-learning’s magnanimity, ease, and convenience of learning from home or the workplace cannot be refuted.^5^ Anderson’s model of e-learning is a true depiction of characteristics of e-learning which are all interdigitated (Fig.1). Still, it is challenging to engage students effectively overall virtual platforms.^6^,^7^

In Pakistan, it was during this pandemic, e-learning emerged as the most innovative teaching and learning methodology. Educational institutes and faculties at all levels were engaged in conventional curriculum modification to an online environment. Despite being the test of agility, this transition to e-learning was hindered in terms of its outcome due to limited access to technology and the internet in remote areas of the country. Although this system gives students the liberty to continue learning without the time and travel constraints^9^,^10^ yet this system was at its infancy in Pakistan so getting acquainted with this system was time-consuming.

With context to all the medical and dental colleges and universities in Pakistan, various studies were conducted to understand the challenges and perception at all levels regarding the transition to e-learning and effective utilization of learning management systems but still, there is a dearth of literature in Khyber Pakhtunkhwa, especially at postgraduate level. Moreover, we need to explore issues in online learning especially in low-income countries where online facilities and resources are already compromised. The purpose of this study was to explore the perception of postgraduate public health students regarding e-learning in context to the Covid-19 pandemic and its effect on their academic performances.

## METHODS

This cross-sectional study was spanned over four months from 3rd October 2020 till 4^th^ February 2021, inducting the post-graduate students already enrolled in the Master of Public Health program at Sarhad Institute of Health Sciences (SIHS) Peshawar. Ethical approval was granted by SIHS (REF NO: SIHS/RandD/ETH/2020/1064).

The Census method was incorporated, and all the 112 students currently enrolled in the MPH program were inducted. Participation in the study was subjected to consent by participants. The confidentiality of all the participants was ensured.

A self-administered questionnaire was designed for data collection through literature search.

Altogether, the questionnaire comprised of 25 items. Four items of the questionnaire covered the demographic details. The questionnaire was further divided into three sections to cover remaining 21 items; (a) to gather information regarding technologies used for e-learning (b) convenience to use e-learning apps (c) to gather information for perception regarding the effectiveness of e-learning. Section b and c of the questionnaire comprise of dichotomous questions. Questionnaire validation was done by two experts in medical education.

The questionnaire was then pilot tested on seventeen participants out of 112, who were not included in the final study. The reliability of the questionnaire (Cronbach alpha) was 0.784. In the final study, 95 students were included. The online questionnaire was circulated among participants via e-mail after taking informed consent. The GPA of all the students was also recorded for conventional on-campus education and after on-line classes during pandemic. All data gathered was recorded and analyzed in SPSS Version-26. Descriptive analysis was done to calculate the mean and frequencies of the data and regression analysis was done to determine the factors predicting the academic performance.

## RESULTS

This study was conducted amongst currently enrolled MPH student’s (n=95), and both male and female students participated in this. In this study, 75.7% of the participants were male (n=72) and 24.2%were female (n=23). Maximum no of participants, 88.4% were in the age group less than 30 years.

Mean GPA of previous semester 1 and semester 2, when they were having a conventional education system on campus before the pandemic was 2.741±0.499 and 2.643±.498 respectively. Since the first semester was newly inducted, they had no previous GPA. The current mean GPA of semesters 1, 2, and 3 who had online classes during this pandemic was 2.41±0.66, 3.06±0.51, and 2.80±0.47 respectively.

Pass percentage was 86.3% (n=82) and 13.7% (n=13) were fail in one or more subjects. The proportion of failure was significantly associated with female gender (26.1) vs 9.7% male (*p* 0.047). Proportion of failure was significantly higher in semester 1 (39.3%) vs 14.3% in semester 2 vs 0% in semester 3 (*p* 0.001).

Table-I reflects the data pertaining to the use of technology including electronic source, internet connectivity mode, and mode of lecture delivery whereas Table-II and Table-III project the data regarding the efficient use of e-learning apps and perception regarding the effectiveness of e-learning.

**Table 1:** Preferred technology for e-learning.

		1^st^ Semester (n=28)	2^nd^ Semester (n=14)	3^rd^ Semester (n=53)	Total
***Electronic source used***					
Categories	Mobile Laptop	19 (67.9%) 9 (32.1%)	2 (14.3%) 12 (85.7%)	30 (56.6%) 23 (43.4%)	51 (53.7%) 44 (46.3%)
***Preferred internet connectivity***					
	Mobile (3G/4G) Wi-Fi Networks	13 (46.4%) 15 (53.6%)	5 (35.7%) 9 (64.3%)	33 (62.3%) 20 (37.7%)	51 (53.7%) 44 (46.3%)
***Mode of Lecture Delivery***					
	Recorded Live	2 (7.1%) 26 (92.9%)	0 (%) 14 (100%)	7 (13.2%) 46 (86.8%)	9 (9.5%) 86 (90.5%)
***Uninterrupted Network***					
	No Yes	5 (17.9%) 23 (82.1%)	5 (35.7%) 9 (64.3%)	21 (39.6%) 32 (6.4%)	31 (32.6%) 64 (67.4%)

**Table II:** Efficient use of e-learning apps.

	1^st^ Semester (n=28)	2^nd^ Semester (n=14)	3^rd^ Semester (n=53)	Total
***Easily use Microsoft Office (Excel, Word, PowerPoint)***				
NO	2 (7.1 %)	2 (14.3%)	6(11.3%)	10 (10.5%)
Yes	26 (92.9%)	12 (85.7 %)	47 (88.7 %)	85 (89.5 %)
***Usage of e-learning apps (zoom, teams, canvas, google classrooms) convenient***				
NO	0 (0%)	2 (14.3%)	9 (17%)	11 (11.6 %)
Yes	28 (100 %)	12 (85.7 %)	44 (83%)	84 (88.4%)
***Able to use learning management system conveniently***				
NO	4 (14.3%)	5 (35.7%)	22 (41.5%)	31 (32.6 %)
Yes	24 (85.7%)	9 (64.3 %)	31 (58.5 %)	64 (67.4%)
***Able to open and use online study material***				
NO	0 (0%)	0 (0 %)	3 (5.7 %)	3 (3.2 %)
Yes	28 (100%)	14 (100%)	50 (94.3%)	92 (96.8%)
***Online material / power point presentations helpful***				
NO	0 (0%)	2 (14.3 %)	1 (1.9%)	3 (3.2 %)
Yes	28 (100%)	12 (85.7 %)	52 (98.1 %)	92 (96.8 %)

**Table III:** Perception regarding the effectiveness of E-learning.

	1^st^ Semester (n=28)	2^nd^ Semester (n=14)	3^rd^ Semester (n=53)	Total
***Prefer online over conventional***				
NO	14 (50%)	6 (42.9 %)	12 (22.6%)	32 (33.7%)
Yes	14 (50 %)	8 (57.1 %)	41 (77.4 %)	63 (66.3%)
***Online teaching method stressful as compared to the conventional teaching method?***				
NO	20 (71.4%)	5 (35.7%)	18(34 %)	43(45.3%)
Yes	8 (28.6%)	9 (64.3%)	35 (66 %)	52 (54.7%)
***Confidently perform online tasks***				
No	0 (0 %)	2 (14.3 %)	12 (22.6%)	12 (22.6%)
Yes	28 (100 %)	12 (85.7%)	41(77.4 %)	41(77.4 %)
***Easily distracted during online sessions***				
NO	24 (85.7%)	6 (42.9 %)	15 (28.3%)	45(47.4 %)
Yes	4 (14.3%)	8 (57.1%)	38 (71.7%)	50 (52.6 %)
***Academic performance positively affected by e-learning***				
NO	9 (32.1%)	2 (14.3%)	15 (28.3%)	26 (27.4%)
Yes	19 (67.9%)	12 (85.7%)	38 (71.7%)	69 (72.6%)
***E-learning effect your health***				
No	27 (96.4 %)	9 (64.3%)	20 (37.7 %)	56 (58.9%)
Yes	1 (3.6%)	5 (35.7%)	33 (62.3 %)	39 (41.1%)
***Communication with the teacher more convenient during online sessions***				
NO	7 (25%)	5 (35.7%)	21 (39.6%)	33 (34.7 %)
Yes	21 (75 %)	9 (64.3 %)	32 (60.4%)	62 (65.3%)
***E-learning is more productive than on-campus learning***				
NO	10 (35.7%)	11 (78.6 %)	33 (62.3%)	54 (56.8%)
Yes	18 (64.3%)	3 (21.4%)	20 (37.7 %)	41 (43.2%)
***E-learning is more compatible with your professional routine?***				
NO	1 (3.6%)	8 (57.1%)	20 (37.7%)	29 (30.5%)
Yes	27 (96.4%)	6 (42.9%)	33 (62.3%)	66 (69.5%)
***Satisfied with the quality of e-learning during the current semester***				
No	1 (3.6%)	7 (50%)	14 (26.4%)	22 (23.2%)
Yes	27 (96.4%)	7 (50%)	39 (73.6%)	73 (76.8%)
*** Family getting socially affected with your e-learning routine***				
NO	24 (85.7%)	11 (78.6%)	30 (56.6%)	65 (68.4%)
Yes	4 (14.3%)	3 (21.4%)	23 (43.4%)	30 (31.6%)
***E-learning should be amalgamated with conventional learning***				
NO	6 (21.4%)	10 (71.4%)	22 (41.5%)	38 (40%)
Yes	22 (78.6%)	4 (28.6%)	31 (58.5%)	57 (60%)

Logistic regression analysis (Table-IV) revealed the age of the student, sex of student, semester, source of learning, access to uninterrupted internet, convenience with LMS, stress, perceived positive impact of e-learning, convenient communication with faculty, the productivity of e-learning compared to on-site learning and compatibility of e-learning with professional routine were not significantly associated with good academic performance (passing all semester subjects) however, students of semester 2 (*p* 0.001) and those using laptops (*p 0.02)* were more likely to get a GPA of 3.0 or above.

**Table IV:** Predictors of Academic Performance.

Parameter	Passed all Subjects		GPA 3.0	

	OR (95% CI)	p value	OR (95% CI)	p value
***Age***				
< 30	***REF***		***REF***	
> 30-45	0.4 (0.1-1.6)	0.177	0.4(-1to1.9)	0.29
***Gender***				
Female	***REF***		***REF***	
Male	3.3 (0.9-11)	0.055	0.4(0.1-0.9)	0.04
***Semester***				
Semester 1	***REF***		***REF***	
Semester 2	3.8 (0.7-20.8)	1.58	5.4(1.3-21.6)	0.01
Semester 3	NA	NA	2.5(0.9-6.8)	0.07
***Source of Learning***				
Mobile/Tablet	***REF***		***REF***	
Laptop/Desktop	1 (0.3-3.3)	0.990	2.6(1.1-6.1)	0.02
***Uninterrupted Internet***				
No	***REF***		***REF***	
Yes	0.9 (0.3-3.2)	0.878	0.8(0.3-1.9)	0.6
***Convenient with LMS***				
No	***REF***		***REF***	
Yes	0.3 (0.07-1.6)	0.170	1.5(0.6-3.6)	0.3
***Stress with online learning***				
No	***REF***		***REF***	
Yes	1.5 (0.4-4.8)	0.505	0.6(0.2-1.4)	0.22
***Perceived positive impact on academics***				
No	***REF***		***REF***	
Yes	1.2 (0.3-4.3)	0.767	1.9(0.8-5)	0.17
***Convenient communication with faculty***				
No	***REF***		***REF***	
Yes	1.2 (0.4-4.0)	0.762	1.2(0.5-2.8)	0.69
***E-learning more productive than on campus***				
No	***REF***		***REF***	
Yes	0.6 (0.2-1.9)	0.405	3.3(1.4-7.9)	0.005
***E-learning compatible with professional routine***				
No	***REF***		***REF***	
Yes	0.4 (0.7-1.8)	0.217	1.3(0.5-3.1)	0.58

## DISCUSSION

The current pandemic has given a new dimension to education in Pakistan when online teaching and learning were successfully incorporated despite being in their infancy. The present study was an attempt to determine the perception of post-graduate students of the Sarhad Institute of Health Sciences regarding e-learning use and effectiveness in learning. The COVID-19 pandemic has made it obligatory for educational establishments all over the world to adjust and implement online teaching for their students.^11^ Before COVID-19, e-learning was almost non-existing in our conventional educational system but, due to nationwide lockdown the institutes had no option but to opt for e-learning, and in a very short time according to higher education guidelines it was started.^12^,^13^

In our study preferred technology for e-learning was mobile with 3,4 G internet connectivity and as students were using mobiles therefore there was an uninterrupted network in most of the instincts. Our findings correlate with other researches where dental and medical undergraduate students have revealed encouraging learning approaches using their smartphones.^14^,^15^ Use of technology especially smartphones and social media for the intention of education is the new trend and is supported by research also where students can improve their learning process and it also complemented the conventional methods.^16^,^17^

Efficient use of e-learning apps like MS Office, zoom, teams, canvas, google Classroom, LMS, and access to online study material/power point presentations showed an overall better response in all students but was more in 3rd-semester students as compare to 1st-semester students. These findings are consistent with studies done in private institutes where due to the availability of resources these institutes were able to develop online teaching material on time as they had both monetary and human resources to embark on this difficult venture.^18^ The reason for better efficiency in 3rd-semester students could be due to their exposure previously to these e-learning apps and during the in-class activity, they are encouraged to use these apps to make their presentations and assignments also.

Student’s perception is important regarding the effectiveness of E-learning over conventional methods. Our findings revealed that the majority of students preferred the online teaching method but found it stressful especially in1st semester as they were not confident in performing online tasks. These findings are compatible with other researches which accounted for varied reactions where some students were in favor of e-teaching and e-learning while others were more comfortable with traditional methods.^19^,^20^

E-learning is compatible with students who are professional which gives them the flexibility to take the class and their work and family life is not affected. This has also been reported in the literature over the conventional methods.^21^ The advantage is that self-directed learning is incorporated in students which is a significant capability in their professional development.^22^,^23^

### Limitation of Study

Since the study includes the students from only one public health institute, the generalizability of result is not possible.

## CONCLUSION

The overall student’s experience in e-learning was satisfactory. However, those having a past understanding of e-learning had higher satisfaction as well as GPA above 3.0 and above. On basis of findings, it is suggested the e-learning is not only the thing of the future but is a need of the day that we can work in partnership with the telecommunication industry to give access to the students with first-class internet coverage at a reasonable price. It is also suggested to conduct a qualitative study to understand and improve the existing e-learning system both for the students and the teachers.

### Authors’ Contribution

**SA** Concept development, tools validation, data collection, data entry, writing first draft of manuscript.

**NM** Tools development and validation, proof reading of manuscript, integrity of research.

**ZK** Literature search, data entry, data analysis, proof reading and writing 2nd draft of manuscript.

**RG** final data analysis, writing of results section of manuscript.

## References

[1] Cucinotta D, Vanelli M (2020). WHO declares COVID-19 a pandemic. Acta Bio Medica:Atenei Parmensis.

[2] Ali N (2020). Students disappointed with online teaching system amid COVID-19.

[3] Kentnor HE (2015). Distance education and the evolution of online-learning in the United States. Curriculum and teaching dialogue.

[4] Anderson J (2005). IT, e-learning and teacher development. Int Educ J.

[5] Vitoria L, Mislinawati M, Nurmasyitah N (2018). Students'perceptions on the implementation of e-learning:Helpful or unhelpful?. Int J Phys:Conference Series.

[6] Iyer P, Aziz K, Ojcius DM (2020). Impact of COVID-19 on dental education in the United States. J Dent Educ.

[7] Desai BK (2020). Clinical implications of the COVID-19 pandemic on dental education. J Dent Educ.

[8] Anderson T (2008). The theory and practice of online learning. Athabasca University Press.

[9] Banditvilai C (2016). Enhancing student's language skills through blended learning. Elec J E-Learn.

[10] Nortvig AM, Petersen AK, Balle SH (2018). A Literature Review of the Factors Influencing E-Learning and Blended Learning in Relation to Learning Outcome, Student Satisfaction and Engagement. Elec J E-Learn.

[11] Salgado H, Castro-Vale I (2020). Clinical communication skills training in dental medical education:the covid-19 pandemic challenge. Healthcare.

[12] Lassoued Z, Alhendawi M, Bashitialshaaer R (2020). An exploratory study of the obstacles for achieving quality in distance-learningduring the COVID-19 pandemic. Educ Sci.

[13] Ansar F, Ali W, Khattak A, Naveed H, Zeb S (2020). Undergraduate students'perception and satisfaction regarding online-learningsystem amidst COVID-19 Pandemic in Pakistan. J Ayub Med Coll Abbottabad.

[14] Zhang C, Fan L, Chai Z, Yu C, Song J (2020). Smartphone and medical application use among dentists in China. BMC Med Inform Decis Mak.

[15] Hamilton LA, Suda KJ, Heidel RE, McDonough SLK, Hunt ME, Franks AS (2020). The role of online-learningin pharmacy education:A nationwide survey of student pharmacists. Curr Pharm Teach Learn.

[16] Senbekov M, Saliev T, Bukeyeva Z, Almabayeva A, Zhanaliyeva M, Aitenova N (2020). The Recent Progress and Applications of Digital Technologies in Healthcare:A Review. Int J Telemed Appl.

[17] Giansanti D (2021). The Artificial Intelligence in Digital Pathology and Digital Radiology:Where Are We?. Healthcare.

[18] Abou El-Seoud M, Taj-Eddin I, Seddiek N, El-Khouly M, Nosseir A (2014). E-learning and students'motivation:A research study on the effect of e-learning on higher education. Int J Emerge technol Learn.

[19] Linjawi AI, Alfadda LS (2018). Students'perception, attitudes, and readiness toward online-learningin dental education in Saudi Arabia:a cohort study. Adv Med Educ Pract.

[20] Arevalo CR, Bayne SC, Beeley JA, Brayshaw CJ, Cox MJ, Donaldson NH (2013). Framework for e-learning assessment in dental education:a global model for the future. J Dent Educ.

[21] Dost S, Hossain A, Shehab M, Abdelwahed A, Al-Nusair L (2020). Perceptions of medical students towards online teaching during the COVID-19 pandemic:a national cross-sectional survey of 2721 UK medical students. BMJ Open.

[22] Cho KK, Marjadi B, Langendyk V, Hu W (2017). Medical student changes in self-regulated learning during the transition to the clinical environment. BMC Med Educ.

[23] Mukhtar K, Javed K, Arooj M, Sethi A (2020). Advantages, Limitations and Recommendations for online-learningduring COVID-19 pandemic era. Pak J Med Sci.

